# Urban health: it's time to get moving!

**DOI:** 10.9745/GHSP-D-14-00071

**Published:** 2014-05-13

**Authors:** Victor K Barbiero

## Abstract

The global health community should mainstream urban health and implement urban health programs to address the triple health burden of communicable diseases, noncommunicable diseases, and injuries in low- and middle-income countries.

## INTRODUCTION

**FIGURE 1.**
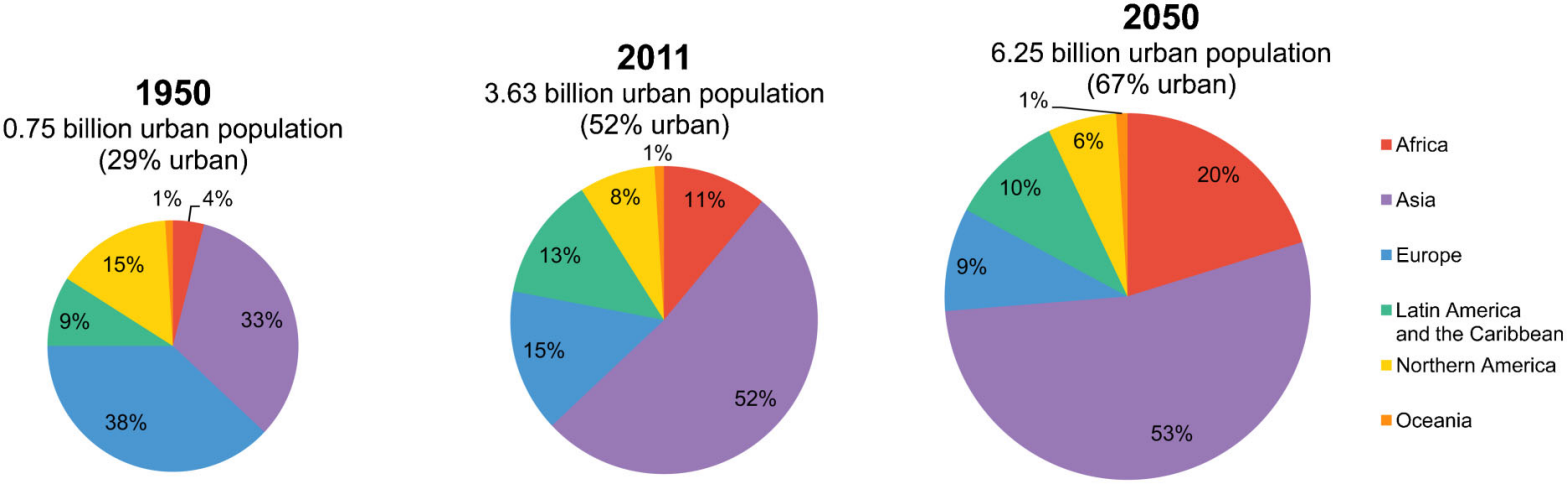
Urban Population Growth by Region Data from the United Nations.^2^

Urbanization is irreversibly increasing around the world. In 2009, the level of urbanization around the world crossed the 50% mark.^1^ By 2050, the world's population will exceed 9 billion and an estimated 67% will live in urban areas (Figure 1).

The United Nations Children's Fund (UNICEF), the World Health Organization (WHO), the U.S. Agency for International Development (USAID), and others portray urbanization as an important priority (Box 1).^3^^–^^5^ However, there is no consolidated global vision, and major investments to address urban health issues, and/or the looming social and environmental threats that urbanization will exacerbate, are few and far between.

**BOX 1. Urban Health Facts**

- The urban transition is here; by 2050, 6.3 billion people will live in urban areas.
- Virtually all of the world's total population growth will be in urban areas of developing countries.
- Most growth is and will be in small and medium-sized cities.
- Megacities (cities with at least 10 million inhabitants) continue to grow.
- Urban slums predominate.
- The urban poor are underserved and underrepresented.
- Poor governance, inequity, social/economic stress, unemployment, and corruption can fuel political unrest across low- and middle-income countries.

Urban growth statistics are from the United Nations.^2^

Between 2011 and 2030, the average annual urban growth rate in low- and middle-income countries is projected to be around 2%,^2^ translating to a doubling of the urban population in 35 years. Approximately 30% to 40% of urban dwellers in low- and middle-income countries live in slums; in Africa, an estimated 62% live in slums.^6^ A great deal of the urban slum population is invisible and/or uncounted.^7^ Their need for water, fuel, power, waste removal, housing, education, health, and employment, and a myriad of other services will be staggering. We are not presently laying the foundations required to deal with the extraordinary physical, social, economic, and epidemiological trends that will characterize the cities of the future. Such neglect will likely adversely affect political stability, disease transmission, social norms, environmental health and safety, and the physical and mental well-being of billions of people.

The time has come to develop a global urban health vision and to commit significant resources to mitigate the adverse health impact of urban growth in low- and middle-income countries. Points of no return have already been passed.

## THE URBAN CRUCIBLE

**FIGURE 2.**
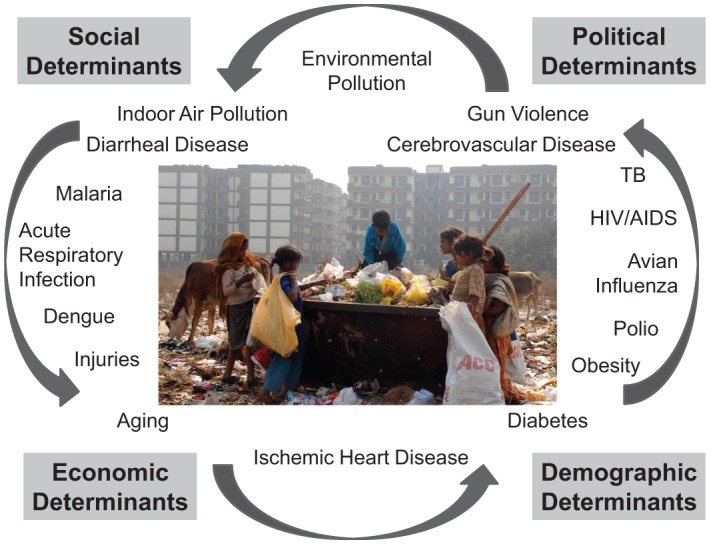
The Urban Crucible Photo credit: ©2008 Pradeep Tewari, Courtesy of Photoshare

The urban environment in low- and middle-income countries represents a crucible in many respects. Varied elements of local ecologies and environments interact forming new cultural, social, demographic, epidemiological, economic, and political processes (Figure 2). Infectious diseases such as diarrhea, respiratory disease, vaccine-preventable diseases, HIV/AIDS, tuberculosis (TB), and vector-borne diseases will continue to persist and spread in urban environs. Additionally, noncommunicable diseases (NCDs) such as ischemic heart disease, stroke, chronic obstructive pulmonary disease, and diabetes will increase. Environmental and social conditions such as indoor/outdoor air pollution, obesity, depression and other mental health issues, vehicular injuries, gang culture, and gun violence will likely also increase, affecting all economic strata. Approximately 54% of disability-adjusted life years globally are due to NCDs, 35% from communicable diseases and maternal, neonatal, and nutritional disorders, and 11% from injuries.^8^ Thus, as global trends indicate, urban populations in low- and middle-income countries face a *triple health burden*, which will be exacerbated in the future.

## THE URBAN OPPORTUNITY

Urbanization also presents new opportunities (Box 2). Population density and closer proximities of health infrastructure could facilitate service delivery by public and private institutions and nongovernmental organizations (NGOs). Mass media through print, radio, and even television have wide audience reach in urban centers. Decentralized authority in urban municipalities could foster pro-poor policies that maximize affordable preventive and curative care for underserved populations. Resources, both public and private, are often greater in the urban environment, and urban centers often enjoy political recognition and support. Furthermore, the commercial sector has providers and products that can be better deployed. The Internet and the ubiquity of mobile phones enhance communication and the potential support for health promotion, disease prevention, and treatment. Perhaps most importantly, urban environs have a middle class and formal sector that support commerce, promote stability, and anticipate change for the better. These groups and cohorts may be early adopters to change.

**BOX 2. Urban Health Opportunities**

- High-density, demarcated populations facilitating outreach for communication, marketing, access, and service delivery
- Independent, concerned municipalities
- Greater access to resources
- More early adopters who are likely to embrace change
- Robust potential for sound public-private partnerships
- New technologies that can enhance health awareness, prevention, and treatment
- Burgeoning middle class to drive policies that benefit themselves and lower-income populations

## CASE STUDIES IN URBAN HEALTH

Although few examples exist that describe remarkable urban health success stories, some notable examples presented below illustrate innovative approaches to urban health issues.

### India: National Urban Health Mission

In May 2013, the Union Cabinet of India approved the National Urban Health Mission (NUMH), clearing the way for implementation of this hallmark program.^10^ The NUMH acknowledges the needs of the urban poor and makes commitments to urban infrastructure and human capacity development. It seeks to integrate existing national services in urban areas, promote equity for slum and other vulnerable populations, decentralize decision-making and implementation to municipalities, and include community organizations in planning. It will provide resources to support 1 urban primary health center per 60,000 people and 1 Auxiliary Nurse Midwife per 10,000 people. Performance-based incentives for public and private providers will aim to improve service delivery. Collaborative public-private health/sanitation/nutrition days will anchor community outreach and encourage service uptake. The Executive Summary for the NUHM Framework aptly describes the spirit of the Mission^9^:

The NUMH will systematically work towards meeting the regulatory, reformatory, and developmental public health priorities of urban local bodies. It will promote convergent and community action in partnership with all other urban area initiatives. Vector control, environmental health, water, sanitation, housing, all require a public health thrust. NUMH will provide resources that enable communitization of such processes. It will provide resources that strengthen the capacity of urban local bodies to meet public health challenges.

The Government of India recognizes that more than 375 million urban dwellers in more than 775 cities represent a development imperative that cannot wait.^9^ Clearly, implementation will be challenging, but the NUHM represents a practical, political, and programming template for other countries.

### Curitiba, Brazil: Sustainable Urban Planning

Curitiba is Brazil's seventh largest city, with a population of about 1.8 million and a doubling time of approximately 39 years.^11^ Despite its size and rapid growth, it is perhaps the most sustainable city in the world.

Since 1965, Curitiba's Master Plan has focused on a cohesive, sustainable urban development strategy that puts the quality of life for its residents at the forefront. A key strategic element of the Master Plan is “radial linear branching,” which governs the placement of new residents and industry along radial axes, expanding outward from the city. These axes are, in turn, served by public transportation, some with bus-only routes.^12^

Curitiba's success is anchored in integrated urban services and an independent planning and implementing agency (the Urban Planning Institute of Curitiba) that sets the vision and coordinates investments. The vision focuses on people-centered efforts that are equitable, sustainable, and ecologically sound. This results in 30% lower use of fuel than other major Brazilian cities, a 45% rate of public transportation use, lower population density (even with an expanding population), cogent land use and zoning, and a network of strategically placed parks and lakes that comprise 20% of the urban landscape.^11^^,^^12^ The parks and lakes were designed to collect flood water, thus reducing flooding and minimizing risk and massive relocations. Residents have planted more than 1.5 million trees,^12^ and they recycle about 13% of solid wastes (compared with only 1% in São Paulo).^11^ Collectively, over time, these actions have increased property values and, consequently, tax revenues. The municipal budget is $600 million per year.^12^

Curitiba has become a highly sustainable city, proving that applying a strategy with a people-first focus and continued, innovative planning can improve the quality of urban life for generations to come. Many lessons reside in the Curitiba example that can be applied elsewhere; the key requirements are sustained political leadership, a long-term vision, an appropriate budget, public commitment, and visible actions that serve the city and its environs (Box 3).

Applying a people-first strategy can improve the quality of urban life.

**BOX 3. Lessons from Curitiba, Brazil**

- Continued political leadership and public commitment
- Creation of an independent planning and implementing agency
- Integrated traffic management, land use, and transportation to promote more pedestrian zones
- Special bus-only avenues with fixed/standard “social” fares that benefit low-income residents
- Flood management through the development of parks and lakes
- “Green Exchange” incentives for garbage disposal (residents get bus tickets or school supplies for dropping off trash at a neighborhood center)
- Recycling: “Garbage that's not garbage program” supports weekly neighborhood collection of recyclable material.
- Open University provides training for residents in vocational skills, crafts, business, management, and environmental conservation.

### Agra and Indore, India: Urban Health Resource Centre's Community-Managed Slum Well-Being Program

The Urban Health Resource Centre (UHRC) is a nonprofit organization in India that focuses on mobilizing slum populations, primarily through support for women's health groups. In Agra, UHRC works in 60 slums and has organized 60 women's groups; in Indore, they work with 90 groups. Every 10–15 groups are assembled into “federations.”^13^^,^^14^ The federations are registered with the government as a formal civil society whose aim is to disseminate health information, promote safe behavior, and effect positive change. The program has improved immunization rates and nutrition for children and has increased antenatal visits, facility deliveries, and breastfeeding levels.^14^ Although sustaining achievements is challenging, the UHRC model has helped to garner NGO support from the Government of India for its “communitization” efforts.^15^ A number of applicable lessons emerge from the UHRC model (Box 4).

**BOX 4. Lessons From Efforts in Agra and Indore, India**

- Promote viable linkages between NGOs and community-based organizations to complement public-sector services and enhance service uptake.
- Map slum facilities and target underserved slum populations.
- Link slum families with existing public- and private-sector services.
- Establish women's groups, cluster coordination teams, and group federations; support these groups with a coordinating NGO.
- Register beneficiaries and unreached families.
- Provide continuous updates to exchange information and encourage continued service outreach.

### New York City: Bloomberg's Health Legacy

U.S. Mayor Michael Bloomberg endeavored to change the health profile of New York City in many ways. He has been praised as an innovator and criticized as a meddler.^16^ Either way, Bloomberg's efforts fundamentally changed policy and public discourse regarding nutrition, physical activity, tobacco, and air pollution.

In 2006, the city required that any food served to customers contain less than 0.5 grams of trans fat per serving. In 2008, the city required all chain restaurants to label menus by disclosing caloric content on menu boards. In 2009, New York launched the National Salt Reduction Initiative (NSRI), a public-private partnership aimed at encouraging companies to reduce sodium content by 20% in overall sales within a given food category such as canned soup. The sugary drink portion limit rule (Portion Cap Rule) proscribed by the Board of Health sparked debate and disapproval locally and nationally. The rule neither bans nor limits the number of sweetened drinks, but limits the size of a drink that can be served by food-serving establishments to 16 ounces. It is mired in the courts, and prospects for approval are likely dim.^16^

Efforts also focused on physical activity and transforming the built environment. New York added almost 400 miles of bicycle lanes, making the city bicycle friendly, and expanded pedestrian access to parks and green spaces. The “High Line” (built on an abandoned elevated rail line) attracts millions of users annually. New York's tobacco control represents a historic effort to reduce smoking and exposure to secondhand smoke. The city's smoke-free law banned smoking in all restaurants and bars, and cigarette taxes increased from US$0.08 to $1.50 per pack. The city also proposed raising the minimum age for buying tobacco and limits on marketing.^16^ Last but not least, the city reinforced an idling law that limits the idling of private vehicles to 3 minutes.

Although New York's health reforms faced, and continue to face, strong challenges from numerous groups, they are applicable to low- and middle-income countries. Clearly, strong leadership is a key requirement to address the political, legal, and civil rights issues surrounding the implementation of urban public health laws.

Strong leadership is key to ensuring innovative, and sometimes politically charged, urban public health laws.

## WORDS TO ACTION

The above examples, albeit important and progressive, do not represent comprehensive approaches to urban health. Major bilateral and multilateral organizations, international NGOs, and foundations should link urban health with all of their major initiatives. HIV prevention and polio eradication efforts already understand the importance of urban areas to their long-term success. Similarly, maternal and child health, reproductive health, TB, malaria, chronic disease, injury, and all other health investments require direct action in urban areas, particularly in slums. Support to urban health efforts can be incorporated relatively easily through the reallocation of existing resources. Working smarter with existing resources should be considered.^17^

By working smarter with existing resources, we can support positive urban health efforts.

Policy makers must commit to a long-term action plan that addresses the triple burden of health issues faced by growing urban populations. A comprehensive global urban health strategy is in order; one similar to the global approach to HIV/AIDS, polio eradication, and malaria. The strategy should build on the urban experience, both positive and negative, from all regions of the globe and provide a clear vision and programmatic guidance. The strategy should include the following general elements:

- Concrete interventions to address the triple health burden of infectious and chronic diseases as well as injuries and trauma
- Use of resources among and between existing global health initiatives
- Expansion of public-private partnerships to capitalize on the existing infrastructure of commercial enterprises in urban areas
- Improved planning, implementation, and management capacity of urban municipalities
- Design and implementation of a set of urban health demonstration programs. These programs should include pillars of technical interventions, policy development, infrastructure support, planning, training, and emergency preparedness with various interventions within each pillar.

The world can ill-afford continued complacency, indecision, or neglect regarding urbanization and urban health.
